# Interrelations of Leptin and Interleukin-6 in Vitamin D Deficient and Overweight Orthodox Nuns from Northern Greece: A Pilot Study

**DOI:** 10.3390/nu17071144

**Published:** 2025-03-26

**Authors:** Spyridon N. Karras, Konstantinos Michalakis, Niki Katsiki, Maria Kypraiou, Antonios Vlastos, Marios Anemoulis, Georgios Koukoulis, Zadalla Mouslech, Filotas Talidis, Georgios Tzimagiorgis, Costas Haitoglou, Μichos Georgios, Evangelos G. Papanikolaou, Skoutas Dimitrios, Neoklis Georgopoulos

**Affiliations:** 1 Laboratory of Biological Chemistry, Medical School, Aristotle University, 54124 Thessaloniki, Greece; tzimagio@auth.gr (G.T.);; 2 Endocrine Practice, Department of Obesity and Metabolism, 11521 Athens, Greece; kostismichalakis@hotmail.com; 3 Department of Nutritional Sciences and Dietetics, International Hellenic University, 57400 Thessaloniki, Greece; nikikatsiki@hotmail.com; 4 School of Medicine, European University Cyprus Nicosia, Nicosia 2404, Cyprus; papanikolaou@assistingnature.gr; 5 Assisting Nature Centre of Reproduction and Genetics, 57001 Thessaloniki, Greece; mariabioanalysis@yahoo.gr; 6 Medical School, Aristotle University, 54124 Thessaloniki, Greecemariosanemoulis@hotmail.com (M.A.); 7 Department of Endocrinology, University of Thessaly School of Medicine, 41500 Larissa, Greece; 8 1st Medical Propedeutic, Department of Internal Medicine, AHEPA University Hospital, Aristotle University of Thessaloniki, 54636 Thessaloniki, Greece; 9 Endocrine Practice, 52100 Kastoria, Greece; 10 Third Department of Obstetrics and Gynecology, Aristotle University of Thessaloniki, 54124 Thessaloniki, Greece; giomichos@hotmail.com; 11 Thermi Clinic, Internal Medicine and Diabetes Department 14th km National Road Thessalonikis-Moudanion, 57001 Thessaloniki, Greece; skoutasd@otenet.gr; 12 Division of Endocrinology, Department of Internal Medicine, School of Health Sciences, University of Patras, 26504 Patras, Greece

**Keywords:** Mediterranean diet, time-restricted eating, Orthodox fasting, leptin, interleukin-6

## Abstract

**Background/Objectives:** Athonian fasting, a rigorous form of intermittent fasting practiced by Christian Orthodox nuns and a subset of the Mediterranean diet, has known health benefits, but its impact on the interplay of adipokines, inflammatory cytokines, and vitamin D status remains under-investigated. This study aimed to elucidate these relationships within this controlled dietary context. **Methods:** This cross-sectional study examined the interplay of leptin, interleukin-6 (IL-6), and vitamin D in 41 overweight, vitamin D-sufficient Christian Orthodox nuns practicing Athonian fasting. Anthropometric, biochemical, and inflammatory markers were assessed in the nuns (mean age 53.4 ± 17.1 years, median monastery stay 17 years, median BMI 26.8 kg/m^2^). **Results:** Analysis revealed significant positive correlations between age and monastery stay (r = 0.615, *p* < 0.001), age and visceral fat (ρ = 0.791, *p* < 0.001), age and IL-6 (ρ = 0.647, *p* < 0.001), and BMI and IL-6 (ρ = 0.622, *p* < 0.001). Strong associations existed between adiposity (BMI, body fat, visceral fat), leptin, and IL-6. Specifically, body fat showed substantial positive correlations with visceral fat (ρ = 0.858, *p* < 0.001), leptin (ρ = 0.538, *p* < 0.001), and IL-6 (ρ = 0.675, *p* < 0.001). Visceral fat demonstrated strong positive correlations with leptin (ρ = 0.613, *p* < 0.001) and IL-6 (ρ = 0.741, *p* < 0.001). A significant positive correlation was also observed between leptin and IL-6 (ρ = 0.507, *p* = 0.003). Conversely, a significant negative correlation was found between 25(OH)D and PTH (ρ = −0.380, *p* = 0.016). Multivariate regression analysis did not reveal independent effects of leptin or IL-6 after adjusting for other factors. **Conclusions:** This study reveals a complex interplay of adiposity, inflammation, and vitamin D status in this unique population of Orthodox monastery fasters. The strong correlations suggest potential targets for interventions aimed at improving metabolic health. Future research should investigate the effects of vitamin D within the context of Athonian fasting.

## 1. Introduction

Obesity and vitamin D deficiency are global health challenges with overlapping metabolic and inflammatory pathways [1,2]. Leptin, primarily secreted by adipose tissue, and interleukin-6 (IL-6), a pro-inflammatory cytokine, play critical roles in energy homeostasis, immune response, and chronic inflammation [3,4,5]. Both biomarkers have been reported to be increased in obesity and implicated in metabolic syndrome, insulin resistance, and cardiovascular disease associated with excess adipose tissue [6,7]. Vitamin D, a fat-soluble vitamin with pleiotropic effects, has been associated with modulating both leptin and IL-6 activity [8,9]. Hypovitaminosis D is common in obesity, possibly due to sequestration of vitamin D stores into adipose tissue or dysregulation of vitamin D equilibrium in people living with obesity [10,11,12]. In this regard, emerging evidence suggests that adequate vitamin D concentrations may mitigate inflammation and leptin resistance, thereby influencing metabolic health [8,9,13]. However, the interplay between leptin, IL-6, and vitamin D homeostasis is largely underexplored in unique populations adopting distinct dietary and lifestyle patterns, like religious Orthodox fasting [14,15]. Previous reports underlined a high prevalence of hypovitaminosis D among Greek religious fasters, particularly those residing in Orthodox monasteries [14,15,16,17,18,19]. Data on the potential biological interactions in similar populations with sufficient vitamin D status remain scarce.

This study focused on Christian Orthodox nuns, a population adhering to Athonian fasting practices characterized by intermittent fasting and high adherence to a vegetarian subtype of the Mediterranean diet. We aimed to explore potential interrelationships between leptin, IL-6, and vitamin D in this group to elucidate potential mechanisms linking dietary patterns, metabolic health, and inflammation.

## 2. Methods

### 2.1. Design

This was a cross-sectional study after a 16-week period of Orthodox religious fasting in a female premenopausal population residing in a monastery in Northern Greece.

### 2.2. Study Population

We recruited a population of Christian Orthodox female adult nuns, 30–50 years of age, residing in Northern Greece.

Orthodox nuns, with a baseline 25-hydroxyvitamin D concentration ≤ 20 ng/mL (as initially evaluated from the same initial cohort—results published previously [16,17,18,19] were excluded). Additional exclusion criteria for both groups were: body mass index (BMI) ≤ 30 kg/m^2^, amenorrhea ≥ 3 months, presence of chronic kidney disease, severe liver disease, diagnosis of prediabetes (fasting glucose 100–125 mg/dL or glycated hemoglobin 5.7–6.4% or blood glucose 140–199 mg/dL at 2 h post 75 g glucose load) or diabetes mellitus (fasting glucose ≥ 126 mg/dL or glycated hemoglobin ≥ 6.5% or blood glucose ≥ 200 mg/dL at 2 h post 75 g glucose load), dyslipidemia, arterial hypertension, or uncontrolled hypothyroidism, recent surgery or severe infections (during the past 3 months), administration of drugs that can alter body weight, glucose and lipid metabolism (e.g., statins, corticosteroids, antipsychotics).

### 2.3. Dietary Patterns

Orthodox nuns followed the Athonian type of fasting as previously described [14,15]. Adherence to dietary plans was evaluated with a 3-day food record at the end of the study, while Nutrition Analysis Software Food Processor 2021 [https://esha.com/products/food-processor/ (accessed on 2 August 2024)] [20] was used to analyze food records. Finally, levels, frequency, and duration of physical activity, divided into light, moderate, and intense physical activity, were recorded for all participants, according to AHA recommendations [21].

### 2.4. Anthropometric Measurements and Biochemical Analysis

Anthropometric measurements and biochemical analyses were performed using standardized procedures. Exact methods, reference ranges, equipment used, and other details were previously analytically described [16,17,18,19]. BMI was calculated as the ratio of weight in kilograms divided by the height in meters squared (kg/m^2^) [22]. In brief, body weight (BW) was recorded to the nearest 0.01 kg using a calibrated computerized digital balance (K-Tron P1-SR, Onrion LLC, Bergenfield, NJ, USA); each participant was barefoot and lightly dressed during measurement. BMI was calculated as the ratio of weight in kilograms divided by the height in meters squared (kg/m^2^) [22]. Body fat (BF) mass and percentage, visceral fat (VF), muscle mass, fat-free mass, and total body water were measured using bioelectrical impedance analysis (SC-330 S, Tanita CorporationBody fat (BF) mass and percentage, visceral fat (VF), muscle mass, fat-free mass, and total body water were measured using bioelectrical impedance analysis [23].

Blood samples were drawn in the morning, after a 12 h overnight fast by antecubital venipuncture, and the samples were stored at −20 °C prior to analysis. Calcium (Ca) concentrations were evaluated using the COBAS8000 automated analyzer system (Roche Diagnostics GmbH, D-68298 Mannheim, Germany). Parathyroid hormone (PTH) and 25(OH)D, were tested in the COBAS e 602 immunochemistry module using electro-chemiluminescence (ECL) technology (Roche Diagnostics GmbH, D-68298 Mannheim, Germany). Reference ranges of values as well as inter- and intra-assay coefficients of variation for the examined parameters are as follows: 25(OH)D: ≥20° ng/mL, 2.2–6.8%, and 3.4–13.1%. Interleukin-6 was measured through the Abcam US Human IL-20R2 ELISA assay, range 7.8–500 pg/mL and sensitivity = 1.6 pg/mL, and leptin through the Abcam US Human Leptin ELISA assay. Sensitivity = 4.65 pg/mL; range = 15.63–1000 pg/mL.

### 2.5. Statistical Analysis

Numerical parameters are presented either as the mean ± standard deviation (SD) or as the median (range) according to their distribution. The Shapiro–Wilk test was used to evaluate normality distribution. Correlations between the studied variables were examined with Pearson or Spearman correlation tests. Multivariate regression analysis was also performed. Statistical significance was defined as a 2-sided *p* value of less than 0.05. We used the SPSS version 22 (IBM Corp: Armonk, NY, USA).

## 3. Results

The study population consisted of 41 Christian Orthodox female adult nuns (mean age 53.4 ± 17.1 years) from two different monasteries in Central and Northern Greece. Median stay in the monasteries was 17 years and median BMI was 26.8 kg/m^2^. Table 1 summarizes anthropometric and laboratory data of the participants. Age was significantly associated with monastery stay (*p* < 0.001), body fat (*p* = 0.006), visceral fat (*p* < 0.001), muscular mass (*p* = 0.018), leptin (*p* = 0.007), and IL-6 (*p* < 0.001). Similarly, monastery stay was significantly related to age (*p* < 0.001), BMI (*p* = 0.003), body fat (*p* = 0.002), visceral fat (*p* < 0.001), leptin (*p* = 0.023), and IL-6 (*p* = 0.024). BMI significantly correlated with monastery stay (*p* = 0.003), body fat (*p* < 0.001), visceral fat (*p* < 0.001), leptin (*p* = 0.017), and IL-6 (*p* < 0.001). Body fat was related to age (*p* = 0.006), monastery stay (*p* = 0.002), BMI (*p* < 0.001), visceral fat (*p* < 0.001), leptin (*p* < 0.001), and IL-6 (*p* < 0.001), whereas visceral fat was associated with age (*p* < 0.001), monastery stay (*p* < 0.001), BMI (*p* < 0.001), body fat (*p* < 0.001), insulin (*p* = 0.008), leptin (*p* < 0.001), and IL-6 (*p* < 0.001).

**Figure 1:**
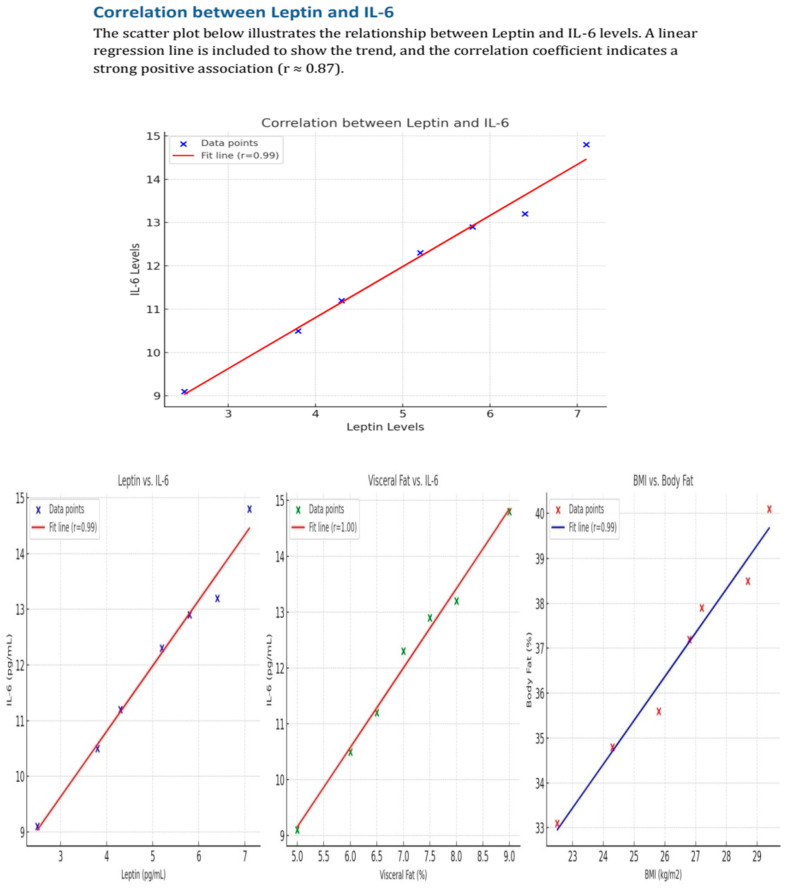
Correlations between leptin, interleukin-6 and visceral fat and body fat.

Insulin correlated to visceral fat (*p* = 0.008), leptin (*p* < 0.001), and IL-6 (*p* = 0.035), whereas 25(OH)D with PTH (*p* = 0.016). Leptin was associated with age (*p* = 0.007), monastery stay (*p* = 0.023), BMI (*p* = 0.017), body fat (*p* < 0.001), visceral fat (*p* < 0.001), insulin (*p* < 0.001), and IL-6 (*p* = 0.003), whereas IL-6 correlated with age (*p* < 0.001), monastery stay (*p* = 0.024), BMI (*p* < 0.001), body fat (*p* < 0.001), visceral fat (*p* < 0.001), insulin (*p* = 0.035), and leptin (*p* = 0.003) (Figure 1). Table 2 summarizes all correlations between the studied variables. Furthermore, multivariate regression analysis did not provide any significant results in relation to associations between leptin or IL-6 and the other variables.

## 4. Discussion

This study examined the interplay of leptin, IL-6, and vitamin D in 41 overweight, vitamin D-sufficient Christian Orthodox nuns practicing Athonian fasting throughout the year. Key findings revealed a strong positive association between visceral fat and both leptin and IL-6. Leptin and IL-6 levels were significantly positively correlated, further supporting their interconnected roles in inflammation and metabolism.

Τhis is the first report of a physiological association of leptin and IL-6 under Athonian fasting conditions and sufficient vitamin D status, which has been reported to affect the interplay of adipokines, particularly in the existence of hypovitaminosis D, which is very common in these populations [24,25].

Findings of this study underscore the complex interactions between leptin, IL-6, and vitamin D in a population with unique dietary practices. Leptin, as a marker of energy homeostasis, is often increased in the presence of excess fat stores [3,4]. IL-6 concentrations are also consistently reported to be increased in similar states and have been associated with chronic inflammation [5,6]. The effects, however, of both nutritional patterns and vitamin D equilibrium on this interplay remain obscure.

Vitamin D has been reported to enhance leptin receptor sensitivity and influence adipose tissue function. In addition, vitamin D exerts rigorous anti-inflammatory properties, particularly through the ability to suppress IL-6 production [26,27].

In our study, attainment of sufficient vitamin D status through a cut-off of 25(OH)D ≥ 20 ng/mL did not affect the synergistic roles of leptin and IL-6, highlighting that interventions targeting vitamin D sufficiency might need different targets for improving metabolic outcomes. We have chosen this threshold for vitamin D sufficiency in this study of physiological associations for several reasons. First, it would be difficult to attain higher vitamin D concentrations in this specific population, with certain sartorial habits, without supplementing with vitamin D, which could affect physiological interactions of adipokines selected. We have previously reported a high prevalence of hypovitaminosis D in Orthodox monastery residents [24,25]. Second, given the small number of participants who accepted to participate in this study, a higher cut-off could significantly reduce the study sample if adopting a higher 25(OH)D as criteria for sufficiency. Third, we believe that this threshold applies a more pragmatic approach for vitamin D status for the Greek general population as well, according to our previous results, without the effects of vitamin D supplementation. However, potential effects of higher attained values of 25(OH)D should be investigated in future supplementation studies.

Available systematic reviews and meta-analyses indicate that vitamin D supplementation is neutral in augmenting leptin concentrations [28], although a few studies demonstrated positive findings [28]. The conflicting effect of vitamin D supplementation on leptin could be partially explained by the variability and lack of adjustments for leptin concentrations in excess fat stores, as well as variability in the methods of measurement, differences in baseline vitamin D status, variability in duration and type, and heterogeneity of dosing regimens, as well as receptor polymorphisms and ethnic differences. On the other hand, leptin has been shown to regulate the expression of vitamin D receptors (VDRs) in various tissues, including adipose tissue, immune cells, and epithelial cells, and can enhance the action of vitamin D in osteoblasts by increasing the expression of vitamin D-dependent genes [29], whereas vitamin D has been reported to act through VDR to inhibit inflammatory pathways and adipokine expression in human adipocytes [30].

Orthodox religious fasting combines characteristics of a vegetarian type of Mediterranean diet and intermittent fasting with feeding time frames of 4–6 h daily. We have previously described the effects of Orthodox religious fasting in serum adipokines in vitamin D-deficient Orthodox nuns and monks, demonstrating no effects of this dietary pattern in adiponectin and leptin concentrations. Results of this study highlight that intermittent Orthodox fasting has no significant effect on adipokine profiles in vitamin D-sufficient populations [25,31,32,33,34].

The consistent interplay of leptin and IL-6 has been previously reported, where leptin induces the secretion of IL-6, particularly in adipocytes and macrophages [35,36,37]. Leptin action is mediated through its receptor (LepR) to initiate signaling pathways that activate pro-inflammatory cytokine release, including IL-6, indicating leptin as a potential mediator linking metabolic status to inflammatory processes. In obesity, where leptin levels are elevated due to increased fat mass and leptin resistance, there is a concomitant increase in IL-6 levels, further contributing to low-grade chronic inflammation that characterizes obesity-related diseases like insulin resistance and cardiovascular disease [35,36,37,38]. Regarding the feedback loop, IL-6 can influence leptin’s action by affecting the sensitivity of leptin receptors. IL-6 can increase the expression of leptin in adipocytes, contributing to the feedback loop between inflammation and adiposity [7,13]. Regarding vitamin D and IL-6, vitamin D has been shown to suppress IL-6 production in various cell types, including immune cells and adipocytes, thus reducing inflammation [35,36]. Moreover, vitamin D inhibits IL-6 production in cancer cells and in endothelial inflammation that sometimes accompanies cardiovascular events [37,38]. At the same time, vitamin D increases anti-inflammatory cytokines, such as IL-10, further contributing to the anti-inflammatory response [39].

Whereas vitamin D has a clear anti-inflammatory effect through suppression of IL-6 secretion, there is no robust biological association with leptin concentrations. A systematic review [8] reported that observational studies indicate an inverse relationship of serum 25(OH)D and leptin concentrations. On the other hand, vitamin D serum concentrations are reduced in excess fat stores due to accumulation and reduced bioavailability, particularly in individuals with increased visceral fat and insulin resistance [10].

Of major interest, increasing 25(OH)D concentrations resulted in a remarkable increase in leptin concentrations [2,3], which highlights that vitamin D supplementation could induce leptinemia. Leptinemia does not seem to be associated with systemic inflammation or insulin resistance development, but limited data indicate that it enhances vitamin D signaling and action [29,30]. It becomes evident that vitamin D at higher doses could restore a favorable cycle of vitamin D and leptin interactions, which, although not evident in this physiological study, could result from higher supplementation regimens, with improved metabolic outcomes. Ensuring adequate vitamin D concentrations, in this regard, might be important for maintaining a healthy immune response and reducing the risk of autoimmune and metabolic diseases.

Religious Orthodox fasting is a vital subset of the Mediterranean diet, practiced by Orthodox populations for spiritual and religious purposes. It combines a vegetarian dietary pattern avoiding meat and dairy consumption and, particularly in Orthodox monasteries, an intermittent feeding pattern, of approximately 16 h of fasting and an 8 h feeding window. The last meal is consumed around 18.30 pm, and the first main meal after early morning ritual ceremonies around 07.00 am, which is consumed in the presence of all residents of the monastery. It typically includes locally cultivated products and also integrates daily physical tasks, necessary for optimal monastery daily life, praying, and spiritual guidance. It usually includes an isocaloric diet (approximately 1400–1600 for nuns), with the exception of specific days (Holy days), where it decreases to around 1200 calories per day. We have excluded similar occasions from our analysis by including a typical Orthodox fasting regimen, as this applies throughout the year in Greek monasteries.

We have previously reported that Greek Orthodox fasting, due to the unique combination of vegetarian and iso- or, in some cases, hypo-caloric patterns and extended daily time-frames of fasting (16–18 h daily), results in significant benefits in adipokine profiles, including increased adiponectin and omentin and reduced visfatin and nesfatin concentrations, compared to standard dietary patterns suggested for dyslipidemia [16,17,18,19,25,31,32,33]. We have also reported favorable effects on glycemic and insulin homeostasis. These results indicate potential favorable cardiovascular effects of this vital subset of the Mediterranean diet.

This study has several limitations, including its cross-sectional design, which limits the ability to establish causal relationships, as well as the relatively small number of participants. We consider, however, that this was a representative sample of Orthodox nuns. In addition, the inclusion of a non-nutritionally restricted control group with vitamin D deficiency might have strengthened the analysis. Finally, since there is only a baseline evaluation, we were unable to establish causal associations. Future longitudinal studies could be required to provide better insights into the cause-and-effect dynamics between these variables, especially regarding Orthodox fasting and vitamin D status.

## 5. Conclusions

This study reveals a complex interplay of adiposity, inflammation, and vitamin D status in a population of Orthodox monastery fasters, without hypovitaminosis D. The strong correlations suggest potential targets for interventions aimed at improving metabolic health. Future research should investigate the effects of vitamin D within the context of Athonian fasting and potential cardiometabolic benefits in the context of vitamin D supplementation.

## Figures and Tables

**Table 1:** Anthropometric and laboratory data of the studied population.

Variables	
Age (years)	53.4 ± 17.1
Monastery stay (years)	17 (1–55)
BMI (kg/m^2^)	26.8 (21.5–48.5)
Body fat (%)	37.2 ± 7.1
Visceral fat (%)	7 (1–21)
Muscular mass (kg)	43.4 (35.3–58.3)
Insulin (μU/mL)	9.44 (2.51–38.45)
Leptin (pg/mL)	19.632 (5.179–75.859
IL-6 (pg/mL)	2.27 (1.0–22.1)
Ca (mg/dL)	9.39 (8.8–11.1)
PTH (pg/mL)	46.4 (26.6–103.0)
25(OH)D (ng/mL)	23.0 ± 9.9

Variables are expressed either as mean ± standard deviation or as median (range) according to their distribution. BMI: body mass index; Ca: calcium; PTH: parathyroid hormone; IL-6: interleukin-6; 25(OH)D: 25-hydroxyvitamin D.

**Table 2:** Correlations between studied variables.

Variables	Pearson (r)/Spearman (rho) Coefficient	*p*
Age		
Age—monastery stay	r = 0.615	<0.001
Age—body fat	r = 0.425	0.006
Age—visceral fat	rho = 0.791	<0.001
Age—muscular mass	rho = −0.368	0.018
Age—leptin	rho = 0.447	0.007
Age—IL-6	rho = 0.647	<0.001
Monastery stay		
Monastery stay—age	r = 0.615	<0.001
Monastery stay—BMI	rho= 0.334	0.003
Monastery stay—body fat	rho = 0.463	0.002
Monastery stay—visceral fat	rho = 0.641	<0.001
Monastery stay—leptin	rho = 0.383	0.023
Monastery stay—IL-6	rho = 0.393	0.024
BMI		
BMI—monastery stay	rho = 0.334	0.003
BMI—body fat	rho = 0.745	<0.001
BMI—visceral fat	rho = 0.696	<0.001
BMI—leptin	rho = 0.399	0.017
BMI—IL-6	rho = 0.622	<0.001
Body fat		
Body fat—age	r = 0.425	0.006
Body fat—monastery stay	rho = 0.463	0.002
Body fat—BMI	rho = 0.745	<0.001
Body fat—visceral fat	rho = 0.858	<0.001
Body fat—leptin	rho = 0.538	<0.001
Body fat—IL-6	rho = 0.675	<0.001
Visceral fat		
Visceral fat—age	rho = 0.791	<0.001
Visceral fat—monastery stay	rho = 0.641	<0.001
Visceral fat—BMI	rho = 0.696	<0.001
Visceral fat—body fat	rho = 0.858	<0.001
Visceral fat—insulin	rho = 0.440	0.008
Visceral fat—leptin	rho = 0.613	<0.001
Visceral fat—IL-6	rho = 0.741	<0.001
Insulin		
Insulin—visceral fat	rho = 0.440	0.008
Insulin—leptin	rho = 0.662	<0.001
Insulin—IL-6	rho = 0.368	0.035
Leptin		
Leptin—age	rho = 0.447	0.007
Leptin—monastery stay	rho = 0.383	0.023
Leptin—BMI	rho = 0.399	0.017
Leptin—body fat	rho = 0.538	<0.001
Leptin—visceral fat	rho = 0.613	<0.001
Leptin—insulin	rho = 0.662	<0.001
Leptin—IL-6	rho = 0.507	0.003
IL-6		
IL-6-age	rho = 0.647	<0.001
IL-6-monastery stay	rho = 0.393	0.024
IL-6- BMI	rho = 0.622	<0.001
IL-6-body fat	rho = 0.675	<0.001
IL-6-visceral fat	rho = 0.741	<0.001
IL-6-insulin	rho = 0.368	0.035
IL-6-leptin	rho = 0.507	0.003
25(OH)D		
25(OH)D—PTH	rho = −0.380	0.016

BMI: body mass index; PTH: parathyroid hormone; IL-6: interleukin-6; 25(OH)D: 25-hydroxyvitamin D.

## Data Availability

The data presented in this study are available on request from the corresponding author.
